# Adaptive Redundant Speech Transmission over Wireless Multimedia Sensor Networks Based on Estimation of Perceived Speech Quality

**DOI:** 10.3390/s110908469

**Published:** 2011-08-31

**Authors:** Jin Ah Kang, Hong Kook Kim

**Affiliations:** School of Information and Communications, Gwangju Institute of Science and Technology (GIST), Gwangju 500-712, Korea; E-Mail: jinari@gist.ac.kr

**Keywords:** wireless multimedia sensor network, speech streaming, packet loss, speech quality estimation, redundant speech transmission, AMR-NB

## Abstract

An adaptive redundant speech transmission (ARST) approach to improve the perceived speech quality (PSQ) of speech streaming applications over wireless multimedia sensor networks (WMSNs) is proposed in this paper. The proposed approach estimates the PSQ as well as the packet loss rate (PLR) from the received speech data. Subsequently, it decides whether the transmission of redundant speech data (RSD) is required in order to assist a speech decoder to reconstruct lost speech signals for high PLRs. According to the decision, the proposed ARST approach controls the RSD transmission, then it optimizes the bitrate of speech coding to encode the current speech data (CSD) and RSD bitstream in order to maintain the speech quality under packet loss conditions. The effectiveness of the proposed ARST approach is then demonstrated using the adaptive multirate-narrowband (AMR-NB) speech codec and ITU-T Recommendation P.563 as a scalable speech codec and the PSQ estimation, respectively. It is shown from the experiments that a speech streaming application employing the proposed ARST approach significantly improves speech quality under packet loss conditions in WMSNs.

## 1. Introduction

Based on advanced technologies for low power and highly integrated digital electronics, wireless sensor networks (WSNs) have emerged and received significant attention as they provide numerous functional applications, e.g., environmental monitoring, human tracking, and military surveillance [1]. Moreover, WSNs have led to another innovation, wireless multimedia sensor networks (WMSNs), which interconnect sensor nodes equipped with multimedia devices such as cameras and microphones [2]. It implies that WMSNs are capable of retrieving audio or video streams, ultimately providing a wide range of potential applications needed to access audio or video data in real-time, not limited to transmitting traditional sensor data.

There have recent reported research studies associated with the implementation methods or the capability analyses for audio or video streaming in WMSNs [3–8]. However, it is hard to guarantee seamless audio or video quality because those multimedia data are typically generated at a much higher bitrates than traditional sensor data. In addition, the reliability of transmission over WMSNs is more degraded than over other networks due to various resource constraints in WMSNs [1,2]. Specifically, packet losses, which are increased due to multi-channel fading, co-channel interference or sensor node failure, become one of the important issues for multimedia streaming applications in WMSNs to meet the quality of service (QoS) requirements [1,3–5]. Therefore, an efficient error protection method to improve the speech quality under packet loss conditions in WMSNs without increasing any network overhead is needed.

**Figure 1.**
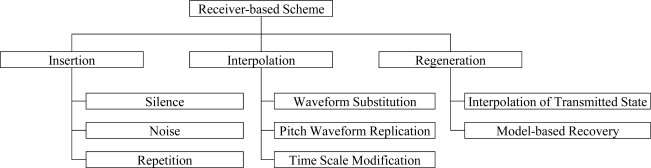
Classification of receiver-based error protection schemes.

**Figure 2.**
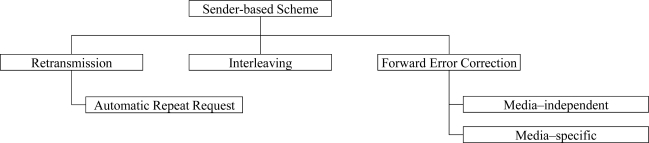
Classification of sender-based error protection schemes.

In order to improve the speech quality in speech streaming applications against packet losses, a number of error protection methods were proposed for IP networks. These methods are typically classified into receiver-based schemes and sender-based schemes, as shown in Figures 1 and 2, respectively [9]. As shown in Figure 1, the receiver-based scheme is a collection of methods that conceal the lost speech signals by using the speech signal characteristics, which is also referred to as packet loss concealment (PLC). That is, the lost speech signals are replaced with silence, noise, or previously reconstructed speech signals [10,11]. This is achieved by interpolating appropriate waveforms from previous and next good speech signals into the lost speech signals [12,13] or regenerating the lost speech signals based on the analysis-by-synthesis criterion of speech signals [14–16].

On the other hand, the sender-based scheme, as shown in Figure 2, tries to protect packet errors by using error-robust transmission methods or by including error correction data. To this end, the lost speech packets are retransmitted [14] or the sequential speech packets are interleaved to avoid burst losses [17]. Moreover, the speech packets are transmitted with forward error correction (FEC) code or redundant data, which are used to recover the lost speech signals at the receiver [18,19]. In addition, robust header compression (ROHC) provides robust speech streaming method on transmission protocol layer, by reducing the overhead due to protocol headers [20].

While the above methods have been proposed for IP networks, several works have also evaluated the performance of the methods under the packet loss conditions in the WMSNs framework. For example, the speech streaming capability over sensor nodes in an operational coal mine [3] was investigated by comparing two waveforms recovered by the receiver-based scheme and the sender-based scheme, respectively. It was revealed in [3] that a speech streaming application employing the receiver-based scheme could accommodate a higher speech coding bitrate under a low packet loss rate (PLR) condition. This led to improved speech quality by using speech streams encoded at a higher bitrate. On the contrary, the sender-based scheme was suitable for dealing with a lower bitrate of speech coding under a high PLR condition in order to assist the speech decoder to recover the lost packets by assigning the remaining bitrate for the redundant data. In addition, a perceptual marking-based error protection method was proposed for the retransmission of speech packets over WMSNs [8]. Here, the PLR experienced by a speech streaming system was effectively reduced by retransmitting speech packets with the help of the cooperative sensor node.

As described so far, there is a trade-off between receiver-based schemes and sender-based schemes in IP networks as well as WMSNs. That is, the receiver-based schemes conceal packet losses without any redundant information from the sender side, yielding transmission bandwidth savings. However, in the receiver-based schemes, the recovered speech quality is usually degraded for the high PLR. On the other hand, the sender-based scheme is more robust for the higher PLR because it can recover packet losses using redundant information given from the sender side, resulting in increased transmission bandwidth. Therefore, an efficient error protection method can be realized by taking advantage of both the receiver-based and the send-band schemes.

In this paper, we propose an adaptive redundant speech transmission (ARST) approach that transmits redundant speech data (RSD) adaptively according to the estimated perceived speech quality (PSQ) and PLR. PSQ is estimated in real-time from speech data received based on a single-ended speech quality assessment. PLR is estimated by monitoring packet loss occurrences from the analysis of real-time transport protocol (RTP) information. In other words, the estimation of PSQ and PLR is based on a receiver-based scheme, while the transmission of RSD is based on a sender-based scheme. In addition, an RTP payload format is suggested as a means of supporting the proposed ARST approach that delivers RSD as well as feedback information in real-time. The effectiveness of the proposed ARST approach is finally demonstrated by using the adaptive multirate-narrowband (AMR-NB) speech codec [21] and ITU-T Recommendation P. 563 [22] as a scalable speech codec and a single-ended speech quality assessment, respectively.

The remainder of this paper is organized as follows. Following this introduction, Section 2 presents the structure of a speech streaming application based on the proposed ARST approach with an RTP payload format. Section 3 describes the proposed ARST approach in detail, and Section 4 discusses the performance of the proposed ARST approach. Finally, this paper is concluded in Section 5.

## 2. A Speech Streaming Application Using the Proposed Adaptive Redundant Speech Transmission

### 2.1. Overview

Speech streaming applications over WMSNs, which are extended from the traditional speech communication services over a public switched telephone network (PSTN) or IP networks, support many useful services such as rescue or military operations where the delivery of speech information in various outdoor environments is needed [1,3]. In particular, a speech streaming node deployed in a WMSN captures speech signals and then segments them into a sequence of speech frames. After that, each speech frame is encoded into a bitstream at a lower bitrate by using a compression algorithm. The speech streaming node packetizes the bitstream followed by transmitting the packetized bitstream using a real-time streaming protocol. At the opposite speech streaming node, the arriving packets are unpacketized into bitstreams and they are decoded into the speech frames, which in turn, are sent to an output device.

**Figure 3.**
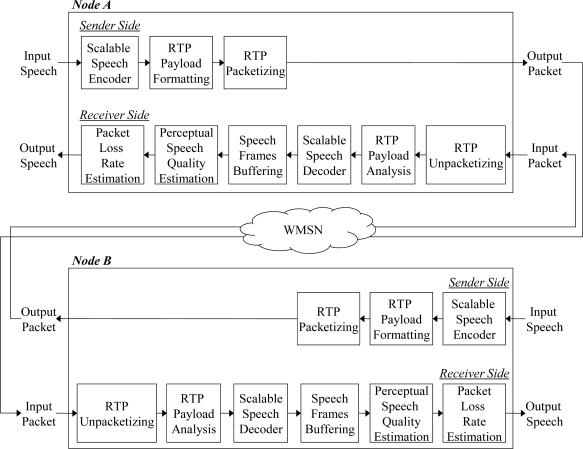
Packet flow for a speech streaming application employing the proposed ARST approach, where *Nodes A* and *B* represent the two communication parties.

Figure 3 shows the packet flow for the speech streaming application simulated in this paper. In the figure, *Nodes A* and *B* represent both parties of the speech stream communication that employ the proposed ARST approach. First, the sender side of *Node A* performs scalable speech encoding for the input speech frame. Next, the sender side generates a packet according to an RTP payload format where the packet includes the current speech data (CSD) bitstream with the decision result for the RSD transmission. The formatted RTP packet is finally transmitted. Note that the RSD bitstream should be incorporated into this payload when the RSD transmission is requested by *Node B*. Meanwhile, after the RTP packet arrives at the receiver side of *Node B*, the receiver side analyzes the received packet from the RTP payload format and then extracts both the CSD bitstream and the decision result for the RSD transmission. In the case that the RTP payload format includes the RSD bitstream, the RSD bitstream is used to recover a lost packet in the future. Next, the extracted CSD bitstream is decoded using a scalable speech decoder and the decoded speech is stored in a speech buffer in order to estimate the PSQ. Finally, the decision result for the RSD transmission according to the estimated PSQ and PLR is comprised to the RTP packet that is sent back to *Node A*.

### 2.2. RTP Payload Format

**Figure 4.**
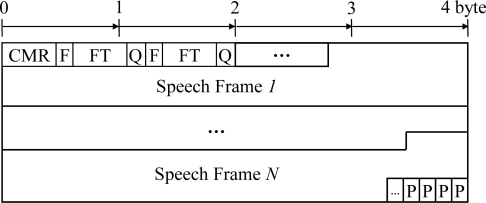
The RTP payload format for AMR-NB speech codec defined in RFC 3267.

As mentioned in Section 2.1, a speech streaming application employing the proposed ARST approach can have an indicator for a scalable bitrate of speech coding. Moreover, in order to deliver the feedback information from *Node A* to *Node B*, or vice versa, there should be a reserved field to accommodate the transmission of the RSD bitstream and feedback information. Thus, we select the RTP payload format defined in IETF RFC 3267 for the AMR-NB speech codec [23], as shown in Figure 4.

In the payload format, the ‘F|FT|Q’ sequence in the control fields is used to describe each speech frame. In other words, a one-bit ‘F’ field indicates whether this frame is to be followed by another speech frame data (F = 1) or if it is the final speech frame data (F = 0). In addition, the FT field, consisting of four bits, then indicates if this frame is actually coded by a speech encoder or if it is a comfort noise. That is, this field is assigned differently from 0 to 7, corresponding to an encoding bitrate of 4.75, 5.15, 5.90, 6.70, 7.40, 7.95, 10.2, and 12.2 kbit/s, respectively. However, if comfort noise is encoded, the assigned number changes from 8 to 11. Note that the number 15 indicates the condition where there is no data to be transmitted, and that the numbers 12 to 14 are reserved for future use. Next, the Q field, indicating the speech quality with one bit, is set to 0 when the speech frame data are severely damaged. Otherwise, it is set to 1. Finally, the codec mode request (CMR) field, consisting of 4 bits, is used to deliver a mode change signal to the speech encoder. For example, it is set to one of eight encoding modes, corresponding to different bitrates of AMR-NB speech codec. At the end of the payload, the ‘P’ field is used to ensure octet alignment. In order to realize the proposed ARST approach with this payload format, two new frame indices for the RSD bitstream and the feedback information are incorporated into the ‘FT’ field, denoted using the numbers 12 and 13, respectively.

The use of the RTP payload format described above has several advantages. First, the control ability for a speech encoder, such as the CMR field, is retained by using the RTP payload format for the speech codec employed in the implemented speech streaming application. Next, the overhead of the control fields for each RSD bitstream is required to be as small as six bits in the ‘F|FT|Q’ field. Finally, no additional transport protocol for the RSD transmission request is needed since this feedback is conducted using the RTP packet that is used to deliver the speech bitstream. Therefore, the transmission overhead for the RSD transmission request is significantly reduced, compared to existing transport protocols designed for feedback such as the RTP control protocol (RTCP) [24].

## 3. Proposed Adaptive Redundant Speech Transmission

### 3.1. Packet Loss Recovery and PSQ Estimation at the Receiver Side

**Figure 5.**
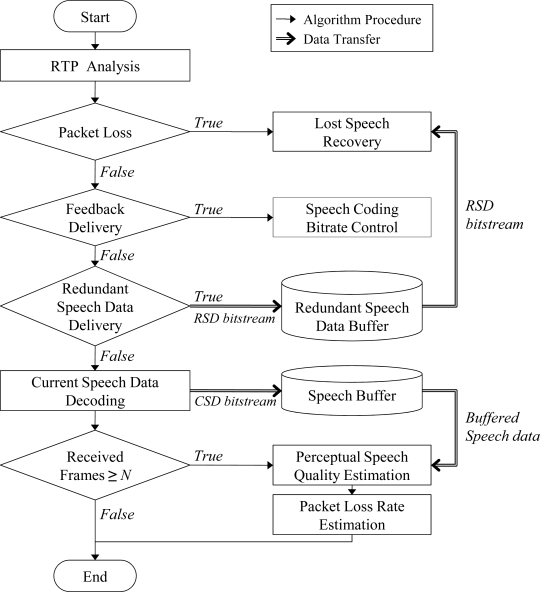
Procedure of the packet loss recovery with the PSQ estimation at the receiver side.

Figure 5 shows the packet loss recovery procedure with the PSQ estimation at the receiver side of a speech streaming node employing the proposed ARST approach. First, a packet loss occurrence is verified through RTP packet analysis. Then, the received CSD bitstream is decoded when there is no packet loss. When a packet loss is declared, the lost speech signals are recovered by using the RSD bitstream or the PLC algorithm employed in a speech decoder, depending on the availability of the RSD bitstream. Finally, the speech decoder reconstructs the speech frame data from the CSD bitstream and estimates the PSQ and the PLR with speech data once the amount of speech frames is enough to estimate a PSQ score.

**Figure 6.**
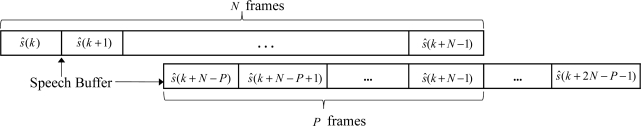
Overlapping structure of speech frames for the PSQ estimation.

In order to estimate PSQ, the speech data of *N* frames are constructed by overlapping with adjacent *P* frames, as shown in Figure 6, where *ŝ*(*k*) is the *k*-th speech frame in a speech buffer. In other words, the PSQ estimation is conducted after (*N – P*) frames are newly received from the opposite speech streaming node. In addition, the estimated PLR, *L̂*(*k*), at the *k*-th frame is obtained by smoothing the previous PLR, *L*(*k* – 1), with the average PLR, *L̄*(*k* – 1), as:

(1) $$\hat{L}(k)=(1-\alpha )\bar{L}(k-1)+\alpha L(k-1)$$

where *α* is a smoothing factor and it is set as 0.4 in this paper from a preliminary experiment to the PLR estimation.

Finally, by comparing the estimated PSQ and PLR with each threshold, it is decided if the request of the RSD transmission is needed. That is, the request for the RSD transmission, *RSD*(*k*), is set to true or false according to Equation (2):

(2) $$\mathrm{RSD}(k)=\{\begin{array}{ll} \mathrm{True}, & \mathrm{if}\;\hat{Q}(k)\leq \theta_{1}\;\mathrm{and}\;\hat{L}(k)\geq \theta_{2}\\ \mathrm{False}, & \mathrm{otherwise} \end{array}$$

where *Q̂*(*k*) is the estimated PSQ score, and *θ*_1_ and *θ*_2_ are thresholds for *Q̂*(*k*) and *L̂*(*k*), respectively.

### 3.2. Scalable Speech Coding and RSD Transmission at the Sender Side

**Figure 7.**
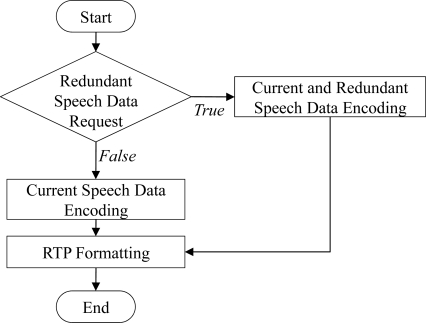
Procedure of transmitting the scalable speech coding bitstream and the adaptive RSD transmission at the sender side.

**Figure 8.**
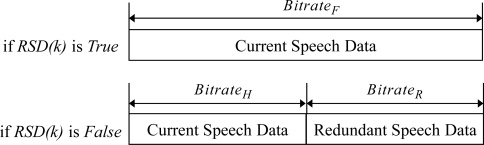
Bitrate assignment according to the RSD transmission.

Figure 7 shows the procedure of transmitting the scalable speech coding bitstream and the RSD bitstream at the sender side for the proposed ARST approach. First, for the received feedback information from the opposite speech streaming node, the sender side verifies the request for the RSD transmission and changes the bitrate of scalable speech coding according to the request. In other words, as shown in Equation (3), when the RSD transmission is not requested, the bitrate, *E_rate_*(*k*), is set to the highest bitrate, *Bitrate_F_*, and then the CSD bitstream is encoded alone with no RSD bitstream (see Figure 8). In other words, *E_rate_* (*k*) is set as:

(3) $$E_{\mathrm{rate}}(k)=\{\begin{array}{ll} {\mathrm{Bitrate}}_{H}, & \mathrm{if}\;\mathrm{RSD}\;(k)=\mathrm{True}\\ {\mathrm{Bitrate}}_{F}, & \mathrm{if}\;\mathrm{RSD}\;(k)=\mathrm{False} \end{array}$$

On the other hand, when the RSD transmission is requested, *E_rate_* (*k*) is set to a smaller bitrate, *Bitrate_H_*, in order to assign the remaining bitrate, *Bitrate_R_*, for the RSD transmission. Thus, both the CSD and RSD bitstream are encoded. Finally, after the RTP payload format described in Section 2.2 is configured according to such adaptive RSD transmission, the RTP packet is transmitted to the opposite speech streaming node. Thus, the speech decoder of the opposite speech streaming node operates at a bitrate, *D_rate_*(*k*), as

(4) $$D_{\mathrm{rate}}(k)=\{\begin{array}{ll} {\mathrm{Bitrate}}_{H}, & \mathrm{if}\;\mathrm{RSD}\;(k)=\mathrm{True}\\ {\mathrm{Bitrate}}_{F}, & \mathrm{if}\;\mathrm{RSD}\;(k)=\mathrm{False} \end{array}$$

As described above, the proposed ARST approach offers several advantages. First, the adaptive operation of the packet loss recovery according to the network condition is effective since the occurrence of packet loss varies; e.g., the PLR varies between 20% and 60% in WSNs [25]. Second, compared to a conventional method that transmits the RSD bitstream for each speech packet by using additional network overhead [9], the proposed ARST approach generates the RSD bitstream without increasing the transmission bandwidth. Third, in order to estimate the network condition, the proposed ARST approach conducts the estimation of PSQ by measuring speech quality.

## 4. Performance Evaluation

### 4.1. Experimental Setup

In order to demonstrate the effectiveness of the proposed ARST approach, a speech streaming application was first implemented by using the AMR-NB speech codec and ITU-T Recommendation P. 563 as a scalable speech codec and a PSQ estimator, respectively. In this work, the speech signals were sampled at 8 kHz, and then encoded using the AMR-NB speech codec operating at 10.2 kbit/s. Thus, when the RSD transmission was needed, the bitrate of the CSD and RSD was set at 4.75 kbit/s each, almost half the bitrate of 10.2 kbit/s. By considering the requirements of ITU-T Recommendation P. 563, *N* was set to 200 frames for the PSQ estimation, which corresponded to 4 s. Moreover, *P* was set to 150 frames, thus the PSQ estimation was conducted when 50 new frames were received.

To compare the speech quality within the same transmission bandwidth, we implemented three conventional error protection methods: an *interleaving approach*, a *fixed redundant speech transmission (RST) approach* and a *PLC approach*. For the interleaving approach, a block interleaver of degree *d* was employed with the permutation defined as as *π*(*id* + *j*) = *i* + *jd*, where *d* = 4 and 0 ≤ *i*, *j* ≤ *d –* 1 [26]. That is, the *i*-th packet, *X_i_*, was re-ordered as *X*_*π*(*i*)_. Note here that AMR-NB was also operated at a rate of 10.2 kbit/s. The fixed RST approach encoded speech signals using the AMR-NB at 4.75 kbit/s with the RSD transmission of 4.75 kbit/s. In other words, the fixed RST approach always transmitted the RSD bitstream for each speech packet, thus the lost speech signals were recovered by using the received RSD bitstream. However, if the RSD bitstream was not received due to burst packet losses, the lost speech signals were then recovered using the PLC algorithm embedded in the AMR-NB decoder. On the other hand, the PLC approach encoded speech signals using the AMR-NB at 10.2 kbit/s without using the RSD transmission. However, the lost speech packets were recovered only by using the PLC algorithm embedded in AMR-NB decoder.

For the following experiments, speech files from the NTT-AT speech database [27] were prepared. Each speech file was about 8 s long, sampled at a rate of 16 kHz. These speech files were filtered using a modified intermediate reference system (IRS) filter followed by an automatic level adjustment [28], and then they were subsequently down-sampled from 16 to 8 kHz.

In order to simulate the packet loss conditions in WMSNs, we used the Gilbert-Elliot channel (GEC) model defined in ITU-T Recommendation G.191 [28]. The GEC model could be considered appropriate for simulating a packet loss environment for WMSNs because of the temporal dependency of packet losses in WMSNs [25,29]. Finally, the packet loss patterns were generated by varying PLR from 3% to 11% by consulting the actual PLRs reported in [3–6,8], and the different patterns were applied for each test. In the work, the mean and maximum burst packet losses (the number of successive packet losses) were measured as 1.5 and 4 packets, respectively.

### 4.2. Threshold Selection for the RSD Transmission

In the proposed ARST approach, the request of RSD transmission was decided according to *θ*_1_ and *θ*_2_ in Equation (2). In this subsection, an experiment was performed to set these thresholds. First of all, in order to find the proper value of *θ*_1_, we measured the average mean opinion score (MOS) for decoded speech by the AMR-NB under no packet loss condition (PLR = 0). To this end, the 24 speech files were used, as described in Section 4.1. As an evaluation method for the recovered speech quality, we used the perceptual evaluation of speech quality (PESQ) defined in ITU-T Recommendation P. 862 [30] due to the following reasons. First, it was shown in that PESQ could provide higher correlation with subjective speech quality than other objective metrics under erroneous packet loss conditions [31,32]. Second, PESQ has been widely used for evaluating speech quality for AMR-NB speech coding under packet loss conditions [33,34]. It was shown from the PESQ measurement experiment that when the estimated MOS was lower than 4.0, speech quality tended to be degraded due to packet losses. Thus, we set *θ*_1_ as 4.0 MOS.

**Figure 9.**
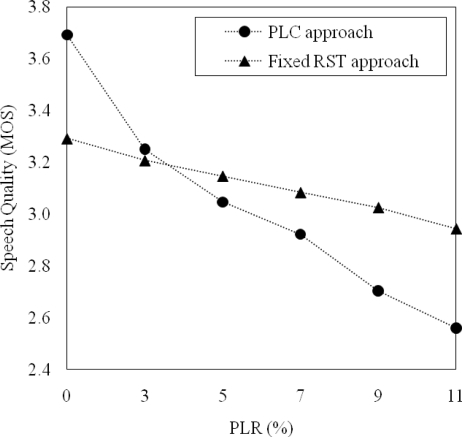
Comparison of speech quality processed by the fixed RST approach and the PLC approach.

The *θ*_2_ in Equation (2) was used to decide when the RSD transmission should be requested. That is, *θ*_2_ could be set by comparing speech quality by the fixed RST approach and that by the PLC approach under different PLR conditions. Figure 9 shows the average MOSs of recovered speech using the fixed RST approach and the PLC approach. It was shown from the figure that the PLC approach improved speech quality more than the fixed RST approach did under the PLRs below 5%. In contrast, the trend of speech quality improvement was reverse depending on PLRs. That is, the fixed RST approach improved speech quality more than PLC approach did under PLRs above 5%. As a result, we set *θ*_2_ as 5% because speech quality could be improved by requesting the RSD transmission when the PLR was higher than 5%.

### 4.3. Performance Evaluation for the Proposed Adaptive Redundant Speech Transmission

In order to demonstrate the effectiveness of the proposed ARST approach, the speech quality of the speech streaming application using the proposed ARST approach was compared to those of the speech streaming applications using the interleaving approach and the fixed RST approach. The speech quality of the speech streaming application using the PLC approach was also evaluated. Note that we also used 28 speech files that were different from those in Section 4.2 but prepared by the same procedure described in Section 4.1.

Table 1 compares the speech quality measured in MOS using PESQ for the different error protection methods under different PLRs ranging from 0% to 11%. As shown in the table, the proposed ARST approach improved the speech quality for low PLRs over other approaches. In addition, the proposed ARST approach provided better performance than the PLC and interleaving approach, as the fixed RST approach did for high PLRs.

Finally, we conducted a statistical analysis by using a one-sided t-test with a 95% confidence level [35] to show how much the proposed ARST approach could improve the MOS scores. First of all, we defined the MOS difference between the proposed ARST approach and a compared one, Δ*MOS*, as Δ*MOS* = *MOS_ARST_*–*MOS_comp_*. It could be declared that the proposed ARST approach was significantly better than the compared one if the following equation was satisfied:

(5) $$\Delta \mathrm{MOS}>t_{\left(1-\alpha ,\nu \right)}S_{0}\sqrt{\frac{1}{n_{\mathrm{comp}}}+\frac{1}{n_{\mathrm{ARST}}}}$$

where *n_comp_* and *n_ARST_* were the numbers of test samples for the compared approach and the proposed ARST approach, respectively. As mentioned earlier, *n_comp_* = *n_ARST_* = 28. In addition, *t*_(1−*α,ν*)_ was the t-statistic with a confidence level of (1 – *α*) when the number of degrees of freedom was *ν* = *n_comp_* + *n_ARST_* – 2. In this paper, *t*_(0.95,*ν*=54)_ ≅ 1.67. In Equation (5), $S_{0}^{2}$ was the pooled estimator of the common variance *S*^2^, which was given by:

(6) $$S_{0}^{2}=\frac{(n_{\mathrm{comp}}-1)S_{\mathrm{comp}}^{2}+(n_{\mathrm{ARST}}-1)S_{\mathrm{ARST}}^{2}}{\left(n_{\mathrm{comp}}+n_{\mathrm{ARST}}-2\right)}$$

where $S_{\mathrm{comp}}^{2}$ and $S_{\mathrm{ARST}}^{2}$ were the sample variances for the compared and proposed ARST approach, respectively.

Table 1 shows average and standard deviation of MOS scores for the different error protection methods under different PLRs ranging from 0 to 11%. In addition, Table 2 shows the MOS difference (MD) and the confidence interval (CI) for the proposed ARST approach against other approaches, where CI was defined as the right term of Equation (5). As described in Table 2, it was seen from the t-test results that the proposed ARST approach significantly improved speech quality under lower PLRs of 3% over all of the other approaches. For high PLRs from 5 to 11%, the proposed ARST approach also significantly improved speech quality over the PLC and interleaving approaches, while it had comparable speech quality to the fixed RST approach.

Finally, we measured the computation complexity of the PSQ estimation in terms of processing time and the percentage of clock speed. For the measurement, we used a laptop platform which was characterized by clock speed of 1.8 GHz and RAM size of 2.0 GB. As a result, it was shown that the PSQ estimation consumed around 0.79 s for test data whose length was 8 s long, thus it occupied less than 9.9% of clock speed. This implies that the proposed ARST approach was expected to operate properly in real-time on sensor node platforms.

## 5. Conclusions

In this paper, we have proposed an adaptive redundant speech transmission (ARST) approach that guarantees speech quality without increasing the transmission bandwidth for speech streaming applications over wireless multimedia sensor networks (WMSNs). To this end, the proposed ARST approach was designed to transmit redundant speech data (RSD) according to the estimation results for the perceived speech quality (PSQ) and the packet loss rate (PLR). Here, a single-ended speech quality assessment and the moving average method were used to estimate the PSQ and the PLR, respectively. The proposed ARST approach was applied to both the receiver side and the sender side of a speech streaming node. The receiver side of the speech streaming node first decided the RSD transmission based on the estimated PSQ and PLR, and then it sent feedback information on the decision result to the opposite speech streaming node via real-time transport protocol (RTP) packets for speech bitstream. On the other hand, the sender side of the speech streaming node controlled the RSD transmission according to the received feedback. The speech coding bitrate was subsequently optimized in order to maintain the equivalent transmission bandwidth despite the RSD bitstream. Finally, we evaluated the speech quality recovered by the proposed ARST approach under different PLRs, and compared it with those of the packet loss concealment (PLC) approach, the interleaving approach, and the fixed redundant speech transmission (RST) approach. It was shown from the results that the proposed ARST approach improved the speech quality as much as 0.139, 0.120, and 0.074 MOS compared to the PLC, interleaving, and fixed RST approach, respectively, under different PLRs ranging from 0% to 11%. This implies that the proposed ARST approach could be applied to speech streaming applications over WMSNs in order to efficiently improve the speech quality degraded due to packet losses.

## Figures and Tables

**Table 1.** Average (Ave.) and standard deviation (SD) of MOS scores measured by PESQ for the different error protection methods, under PLRs ranging from 0% to 11%.

**PLR (%)**	**PLC Approach**		**Interleaving Approach**		**Fixed RST Approach**		**Proposed ARST Approach**	
	**Ave.**	**SD**	**Ave.**	**SD**	**Ave.**	**SD**	**Ave.**	**SD**
0	3.676	0.085	3.676	0.075	3.255	0.116	3.676	0.085
3	3.292	0.132	3.291	0.122	3.187	0.157	3.362	0.142
5	2.946	0.126	2.951	0.132	3.058	0.130	3.008	0.121
7	2.849	0.181	2.893	0.180	3.050	0.151	2.989	0.160
9	2.757	0.184	2.789	0.193	3.012	0.145	3.002	0.142
11	2.656	0.162	2.692	0.156	3.002	0.164	2.972	0.156

**Table 2.** Statistical test results of the ARST approach against each of the compared approaches such as PLC, interleaving, and fixed RST approach under different PLRs ranging from 0% to 11%, where MOS difference (MD) and confidence interval (CI) for each PLR condition were also shown.

**PLR (%)**	**PLC Approach**		**Interleaving Approach**		**Fixed RST Approach**	
	**MD**	**CI**	**MD**	**CI**	**MD**	**CI**
0	0.000	0.038	0.000	0.036	0.421	0.046
3	0.070	0.061	0.070	0.059	0.176	0.067
5	0.062	0.055	0.062	0.057	−0.050	0.056
7	0.140	0.076	0.140	0.076	−0.060	0.069
9	0.245	0.073	0.245	0.076	−0.010	0.064
11	0.316	0.071	0.316	0.070	−0.030	0.072
